# The impact of the interaction between ovarian markers and the endometrium on the outcomes of assisted reproduction

**DOI:** 10.3389/fcell.2026.1725501

**Published:** 2026-05-01

**Authors:** Xuefei Li, Zhonghua Ai, Yan Jia, Hai Li, Qun Lv, Lili Wang

**Affiliations:** 1 Department of Reproductive Medicine, Sichuan Jin’xin Xi’nan Women’s and Children’s Hospital, Chengdu, Sichuan, China; 2 Department of Obstetrics and Gynecology, The Affiliated Hospital, Southwest Medical University, Luzhou, Sichuan, China; 3 Department of Epidemiology, School of Public Health, Sun Yat-sen University, Guangzhou, China; 4 The People’s Hospital of Fengqing, Lincang, Yunnan, China; 5 Department of Obstetrics and Gynecology, Sichuan Provincial People’s Hospital, Chengdu, Sichuan, China

**Keywords:** AFC, AMH, clinical pregnancy rate, endometrial thickness, live birth rate

## Abstract

**Objective:**

To investigate the interaction effects of anti-Müllerian hormone (AMH), antral follicle count (AFC), basal follicle-stimulating hormone (FSH), and endometrial thickness on the outcomes of assisted reproductive technology (ART).

**Methods:**

This retrospective cohort study included 12,071 infertile patients with endometrial thickness of 7–14 mm who underwent fresh embryo transfer at a large reproductive medical center in Chengdu, China, between 2019 and 2022. Optimal thresholds for AMH, AFC, and basal FSH were determined using receiver operating characteristic (ROC) curve analysis, and patients were subsequently stratified into relatively normal and abnormal ovarian response groups based on these cut-offs. Cox proportional hazards models were employed to assess the association between endometrial thickness and pregnancy outcomes, including biochemical pregnancy, clinical pregnancy, and live birth. Stratified analyses and interaction tests were further conducted to evaluate the modifying effects of AMH, AFC, and basal FSH levels on this association. Sensitivity analyses were performed to verify the robustness of the findings.

**Results:**

Multivariable-adjusted Cox regression indicated that among patients with AMH ≥2.59 ng/mL or AFC ≥11, each 1-mm increase in endometrial thickness was associated with modest outcome improvements: a 1.5% increase in biochemical pregnancy rate [hazard ratio (HR) 95% confidence interval (CI): 1.015 (1.002, 1.029)], a 0.8% increase in clinical pregnancy rate [HR 95% CI: 1.008 (1.001, 1.015)], and a 0.7% increase in live birth rate [HR 95% CI: 1.007 (1.001, 1.013)]. Baseline FSH levels showed no significant association with pregnancy outcomes. Statistically significant interactions were observed between endometrial thickness and both AMH and AFC (*P*-interaction < 0.05). Sensitivity analyses supported the robustness of these associations.

**Conclusion:**

Among patients with normal ovarian reserve, endometrial thickness within the range of 7–14 mm showed a positive albeit modest association with ART pregnancy outcomes, with AMH and AFC levels exerting a modifying effect on this association.

## 1 Introduction

Infertility has become one of the most common global health challenges, affecting an estimated 186 million individuals worldwide, which corresponds to approximately 17% of all couples (Inhorn and Patrizio, 2015). Assisted reproductive technology (ART) serves as a primary and effective therapeutic intervention for infertility and has advanced rapidly in recent years. Data from 2023 on medically assisted reproduction (MAR) in European countries reflect a continuous rise in ART-associated live birth rates (Smeenk et al., 2023). Critical determinants of *in vitro* fertilization (IVF) outcomes include female age, anti-Müllerian hormone (AMH), follicle-stimulating hormone (FSH), antral follicle count (AFC), embryo quality, and endometrial status (Vaegter et al., 2017). Still, researchers are working to define the exact role and weight of each factor, and opinions vary. For instance, Iliodromiti et al. identified AFC as an independent risk factor for ovarian hyperstimulation (Iliodromiti et al., 2015), whereas Fang et al. highlighted basal FSH as a key predictor of ovarian response (Fang et al., 2015). Accumulating evidence further suggests that serum AMH may hold superior predictive value for ART outcomes compared to other biomarkers (Bedenk et al., 2020; Bell et al., 2021; Nelson et al., 2023).

The endometrium serves as the foundation for embryo implantation and development, and its thickness is a key indicator for predicting pregnancy outcomes (Fu et al., 2024). However, there is no consensus on the effect of endometrial thickness on assisted reproductive outcomes. A multicenter retrospective cohort study comprising 30,676 cycles across 25 IVF centers in 3 countries reported that an endometrial thickness of <7 mm was associated with a lower live birth rate (Genovese et al., 2025). Huang et al. analyzed 25,683 fresh *in vitro* fertilization-embryo transfer (IVF-ET) cycles and reported a peak in live birth rates at an endometrial thickness around 12 mm (Huang et al., 2025). In contrast, a meta-analysis showed that endometrial thickness alone cannot serve as an independent predictor of IVF outcomes (Kasius et al., 2014). Similarly, Yang et al. reported that neither endometrial thickness nor morphology, alone or in combination, can accurately predict live birth following IVF (Yang et al., 2018). Therefore, some researchers have begun to combine endometrial parameters with other markers to assess their collective impact on assisted reproductive outcomes. For example, Lv et al. demonstrated that integrating embryo quality with endometrial factors can improve the prognostic value for live birth in patients undergoing fresh embryo transfer (Lv et al., 2020).

However, the interaction between endometrial thickness and varying levels of AMH, AFC, and FSH has rarely been examined. This study aims to both examine the moderating effects of these ovarian reserve markers and evaluate their combined predictive value for IVF pregnancy outcomes.

## 2 Materials and methods

### 2.1 Study population

**FIGURE 1:**
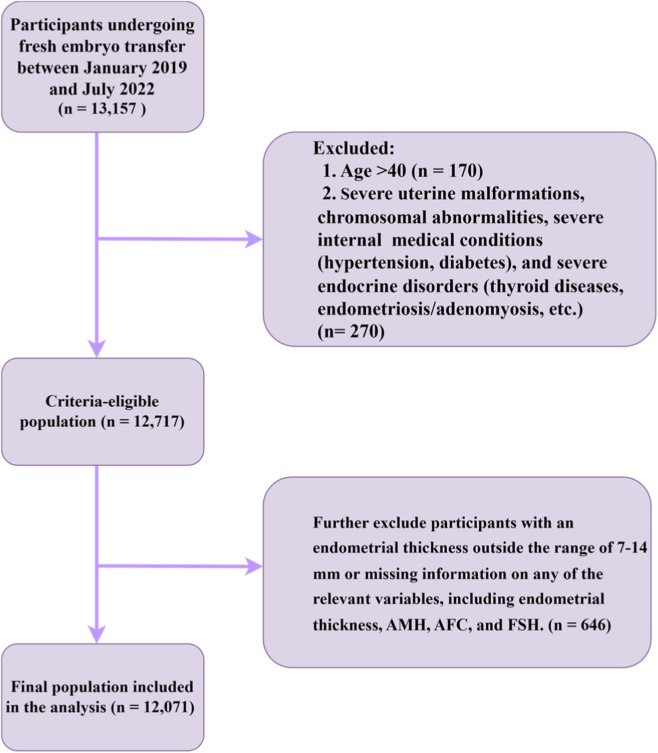
Patient inclusion flowchart. AMH, Anti-mullerian hormone; AFC, Antral Follicle Count; FSH, Follicle Stimulating Hormone.

In this retrospective cohort study, we included 13,157 infertile patients who underwent fresh embryo transfer at Sichuan Jin’xin Xi’nan Women and Children’s Hospital in China between 7 January 2019, and 5 July 2022. The study aimed to examine the relationship between AMH, AFC, basal FSH, and endometrial thickness with assisted reproductive technology outcomes. Participants aged over 40 years were excluded. Subsequently, patients with severe uterine anomalies, chromosomal abnormalities, major systemic disorders (e.g., hypertension and diabetes), significant endocrine diseases (including thyroid disorders, endometriosis, and adenomyosis), or other relevant conditions were also excluded. Previous studies have shown that the association between endometrial thickness and pregnancy prognosis follows a continuous gradient, making it difficult to define a universal critical threshold for embryo transfer (Pérez-Milán et al., 2025). However, a thin endometrium (<7 mm) is strongly correlated with a significantly reduced pregnancy rate (Genovese et al., 2025; Song et al., 2023; Wang et al., 2024; Ye et al., 2025), while an excessively thick endometrium (>14 mm) may lead to diminished pregnancy efficacy and an elevated risk of miscarriage (Weissman et al., 1999; Xu et al., 2021). To mitigate potential confounding effects of extreme endometrial thickness on pregnancy outcomes, patients with endometrial thickness <7 mm or >14 mm were excluded. Finally, patients lacking any of the following parameters (endometrial thickness, AMH, AFC, or basal FSH) were also excluded from the study (Figure 1). Each couple involved in the study provided informed consent to receive assisted reproductive treatment. The study was approved by the Ethics Committee of Sichuan Jin’xin Xi’nan Women and Children’s Hospital (IRB 2025–014) and was conducted in accordance with the principles of the Declaration of Helsinki.

### 2.2 Hormone and ultrasound measurements

All subjects had their hormone levels and AFC measured between days 2 and 5 of their menstrual cycles. Serum levels of AMH and FSH were measured using chemiluminescent immunoassays on a Beckman Coulter Access immunoassay analyzer (Access AMH assay and Access FSH assay, respectively). A 7-MHz transvaginal ultrasound probe was employed for real-time two-dimensional imaging to count antral follicles within the ovaries. Follicles with diameters ranging from 2 to 9 mm were included in the AFC count (Coelho Neto et al., 2018). On the day of human chorionic gonadotropin (hCG) administration, endometrial thickness was measured in the mid-sagittal plane of the uterine body.

### 2.3 Assisted reproductive techniques

The ovarian stimulation protocols used included the gonadotropin-releasing hormone (GnRH) antagonist protocol, GnRH agonist protocol, and mild-stimulation protocol. These have been described in detail previously (Oehninger, 2011). Once three or more follicles attained a diameter greater than 17 mm, hCG, GnRH agonist (GnRH-a), or a dual-triggering strategy was implemented to facilitate the final maturation of the follicles. Oocyte retrieval was carried out 36–38 h subsequent to the triggering event. When the number of retrieved oocytes exceeded 20 or other factors potentially associated with the risk of ovarian hyperstimulation were present, the entire embryo cryopreservation approach was selected for that particular cycle. Eligible patients underwent embryo transfer on either day 3 or day 5 after oocyte retrieval, followed by routine luteal phase support.

### 2.4 Primary outcome measures

All participants were followed from the date of fresh embryo transfer until the conclusion of pregnancy (Ai et al., 2025). The primary outcome was live birth, while biochemical and clinical pregnancies were assessed as secondary outcomes. Biochemical pregnancy was defined as a serum β-hCG level >25 mIU/mL measured 10–14 days after embryo transfer, with a negative result indicating the non-occurrence of this event. Clinical pregnancy was defined as the confirmation of an intrauterine gestational sac via ultrasonography following embryo transfer; failure to confirm this finding was regarded as the non-occurrence of clinical pregnancy. Live birth was defined as the delivery of a fetus exhibiting any evidence of life (e.g., respiration, heartbeat). In the analysis of the live birth endpoint, the occurrence of adverse pregnancy outcomes such as miscarriage or stillbirth during the follow-up period was deemed equivalent to the non-occurrence of the live birth event. Furthermore, the assessment and coding of each endpoint adhered to the temporal logic inherent in pregnancy progression: if a preceding endpoint did not occur, all subsequent endpoints were automatically recorded as non-occurring.

### 2.5 Statistical analysis

In light of the absence of internationally recognized cut-off values for AMH, AFC and FSH, we employed receiver operating characteristic (ROC) curve analysis, with the maximization of Youden’s index as the criterion, to determine the optimal discriminatory thresholds for each indicator. Patients were then stratified into a relatively normal ovarian response group and a relatively abnormal ovarian response group based on these thresholds.

A Cox proportional hazards regression model was utilized to estimate the hazard ratios (HRs) and their 95% confidence intervals (CIs) for the associations between endometrial thickness and biochemical pregnancy, clinical pregnancy, and live birth rates in the context of assisted reproduction.

Based on current knowledge of assisted reproduction and findings from prior studies (Carson and Kallen, 2021; Lawrenz and Fatemi, 2022), we constructed two statistical models. Model 1 was adjusted for female age and ethnicity. Building upon Model 1, Model 2 further incorporated the following variables: ovarian stimulation protocol, total gonadotropin (Gn) dosage, estradiol (E2) level, endometrial pattern on the day of hCG administration, body mass index (BMI), duration of infertility, prolactin level, number of high-quality cleavage-stage embryos transferred, number of high-quality blastocyst-stage embryos transferred, male sperm concentration, smoking status, blood glucose level, triglyceride level, total cholesterol level, and low-density lipoprotein cholesterol (LDL-C) level. High-quality cleavage-stage embryos are defined as ≥7 cells on day 3 with ≤20% fragmentation and high-quality blastocyst-stage embryos are defined as Gardner grade ≥3BB.

To investigate whether AMH, AFC, and basal FSH levels could modulate the relationships between endometrial thickness and the biochemical pregnancy rate, clinical pregnancy rate, and live birth rate, we conducted stratified analyses according to different levels of AMH, AFC, and basal FSH. Additionally, we explored the impact of the interactions among AMH, AFC, basal FSH, and endometrial thickness on the outcomes. Specifically, by adjusting for confounding factors within Model 2 using the cross-product terms of different hormone levels and endometrial thickness, we examined the interactions among AMH, AFC, basal FSH, and endometrial thickness. More precisely, we compared the −2 log-likelihood values of the models with and without the cross-product terms of AMH, AFC, FSH, and endometrial thickness to assess the influence of these interaction terms on the model’s goodness-of-fit.

### 2.6 Sensitivity analyses

We conducted a series of sensitivity analyses. First, considering the potential influence of an extended duration of infertility on the study results, we excluded 280 patients whose infertility duration exceeded 10 years. Consequently, 11,791 participants were included in this replicated analysis. Second, for the missing data of study variables such as endometrial thickness, AMH, AFC, and FSH, we employed multiple imputation techniques; consequently, all associations were evaluated in the full cohort of 12,717 participants. Third, to assess threshold robustness, this study conducted directional adjustments and analyses of the indicators. The cut-off values for the positive indicators AMH and AFC were each lowered by 10% to 2.33 ng/mL and 10 follicles respectively, while the cut-off value for the negative indicator basal FSH was raised by 10% to 9.58 mIU/mL. Finally, a stratified analysis was conducted by age (using 35 years as a cutoff) to validate the robustness of the conclusions.

All statistical analyses were performed using R software (version 4.4.1). A *p*-value of less than 0.05 was regarded as statistically significant.

## 3 Results

### 3.1 Characteristics of the study population and study grouping

In the final analysis sample consisting of 12,071 individuals, the mean (SD) age was 30.87 (4.01) years. Among them, 91.83% were of Han ethnicity, and 43.80% had an educational attainment of “Specialist or bachelor’s degree” (Table 1).

**TABLE 1:** Descriptive characteristics of participants with different assisted conception outcomes.

Characteristics	Infertility patients (N = 12,071)	Biochemical pregnancy (N = 7,300)	Clinical pregnancy (N = 6,320)	Live birth (N = 5,324)	*p*-value
Female age (years), mean (SD)	30.87 (4.01)	30.62 (3.85)	31.55 (3.76)	30.31 (3.70)	<0.001
Female ethnicity, n (%)	​	​	​	​	<0.001
Han ethnicity	11,085 (91.83)	6745 (92.40)	5852 (92.59)	4935 (92.69)	​
Ethnic minority	986 (8.17)	555 (7.60)	468 (7.41)	389 (7.31)	​
Female education level, n (%)	​	​	​	​	0.515
Specialist or bachelor’s degree	5288 (43.80)	3256 (44.60)	2832 (44.81)	2396 (45)	​
Master’s degree and above	338 (2.80)	219 (3)	189 (3)	159 (2.99)	​
Other	6445 (53.40)	3825 (52.40)	3299 (52.19)	2769 (52.01)	​
Male smoking status	​	​	​	​	0.996
No	11,590 (96.10)	7023 (96.20)	6080 (96.20)	5122 (96.21)	​
Yes	481 (3.90)	277 (3.80)	240 (3.80)	202 (3.79)	​
Body mass index (kg/m^2^), mean (SD)	22.21 (3.10)	22.20 (3.10)	22.20 (3.09)	22.17 (3.05)	<0.001
Blood glucose (mmol/L), mean (SD)	5.21 (0.58)	5.22 (0.58)	5.22 (0.58)	5.21 (0.56)	<0.001
Triglycerides (mmol/L), mean (SD)	1.22 (0.95)	1.20 (0.95)	1.22 (0.95)	1.21 (0.91)	<0.001
Total cholesterol (mmol/L), mean (SD)	4.35 (0.78)	4.34 (0.77)	4.33 (0.78)	4.35 (0.79)	<0.001
Low-density lipoprotein cholesterol(mmol/L), mean (SD)	2.43 (0.66)	2.43 (0.65)	2.43 (0.65)	2.44 (0.65)	<0.001
Progesterone (ng/mL), median (IQR)	0.61 (0.34)	0.62 (0.35)	0.61 (0.33)	0.60 (0.34)	<0.001
Prolactin (ng/mL), median (IQR)	253.73 (197.65)	253.75 (181.36)	253.73 (176.180)	252.72 (174.83)	<0.001
Luteinizing hormone (mIU/mL), median (IQR)	4.38 (3.06)	4.25 (2.995)	4.36 (3.20)	4.41 (2.93)	<0.001
Sperm concentration (millions/mL), median (IQR)	54.00 (63.00)	54.00 (63.00)	53.00 (62.00)	53.00 (63.00)	<0.001
Endometrial thickness (mm), mean (SD)	10.56 (1.92)	10.58 (1.93)	10.58 (1.93)	10.60 (1.92)	<0.001
AMH (ng/mL), n (%)	​	​	​	​	<0.001
Normal	7064 (58.50)	4477 (61.30)	3880 (61.40)	3292 (61.80)	​
Abnormal	5007 (41.50)	2823 (38.70)	2440 (38.60)	2032 (38.20)	​
FSH (mIU/mL), n (%)	​	​	​	​	0.829
Normal	8790 (72.80)	5376 (73.60)	4634 (73.30)	3897 (73.20)	​
Abnormal	3281 (27.2)	1924 (26.40)	1686 (26.70)	1427 (26.80)	​
AFC, n (%)	​	​	​	​	0.860
Normal	8530 (70.70)	5309 (72.70)	4605 (72.90)	3885 (73.0)	​
Abnormal	3541 (29.30)	1991 (27.30)	1715 (27.10)	1439 (27.0)	​
Years of infertility (years), mean (SD)	3.55 (2.75)	3.48 (2.63)	3.47 (2.64)	3.42 (2.55)	<0.001
hCG day estradiol (pg/mL), median (IQR)	2316.42 (1132.65)	2307.65 (1098.14)	2286.56 (1086.58)	2274.1 (1072.21)	<0.001
Total gonadotropin dose (IU), median (IQR)	1950 (900)	1950 (900)	1950 (900)	1925 (900)	<0.001
Endometrial pattern, n%	​	​	​	​	<0.001
Homogeneous	11,751 (97.36)	7154 (97.28)	6153 (97.35)	5179 (97.26)	​
Triple-line	320 (2.65)	198 (2.72)	167 (2.64)	145 (2.72)	​
Number of high-quality cleavage-stage embryos transferred, median (IQR)	1.00 (1.00,2.00)	1.00 (1.00,2.00)	2.00 (1.00,2.00)	2.00 (1.00,2.00)	<0.001
Number of high-quality blastocysts transferred, median (IQR)	1.00 (1.00,2.00)	1.00 (1.00,2.00)	1.00 (1.00,2.00)	1.00 (1.00,2.00)	<0.001
Treatment protocol, n (%)	​	​	​	​	<0.001
GnRH agonist	6280 (52.02)	4182 (57.29)	3640 (57.59)	3097 (58.17)	​
GnRH antagonist	5580 (46.23)	2991 (40.97)	2580 (40.82)	2132 (40.84)	​
Other protocols	211 (1.75)	127 (1.74)	100 (1.58)	95 (1.78)	​

Abbreviations: Continuous variables were tested by analysis of variance, and categorical variables were tested by chi-square test. SD, standard deviation; IQR, interquartile range; AMH, Anti-mullerian hormone; AFC, antral follicle count; FSH, Follicle Stimulating Hormone. GnRH, Gonadotropin-Releasing Hormone.

**FIGURE 2:**
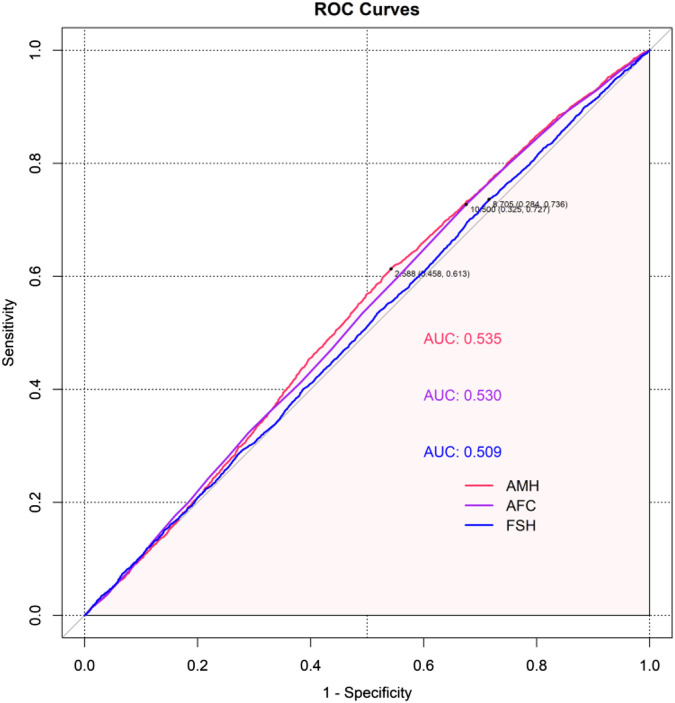
ROC curves for predicting live births using AMH, AFC and basal FSH. AMH, Anti-mullerian hormone; AFC, Antral Follicle Count; FSH, Follicle Stimulating Hormone. The red, purple and blue curves in the figure represent the ROC curves for AMH, AFC and basal FSH respectively. The sensitivity and specificity corresponding to the optimal cut-off values for each indicator are indicated in brackets. The area under the curve reflects the overall predictive performance of the respective indicator.

In this study, optimal discriminatory thresholds for AMH, AFC, and basal FSH were determined using ROC curve analysis, and patients were subsequently stratified into normal and abnormal ovarian response groups based on these thresholds. The results indicated optimal cut-off values of 2.59 ng/mL for AMH, 11 for AFC, and 8.70 mIU/mL for basal FSH (Figure 2). Based on these thresholds, patients meeting any of the following criteria were classified into the relatively normal ovarian response group: AMH ≥2.59 ng/mL, AFC ≥11, or basal FSH ≤8.70 mIU/mL. All other patients were categorized into the relatively abnormal ovarian response group.

### 3.2 Association between endometrial thickness, AMH, AFC, FSH, and outcomes

During the follow-up period of 4342.53 person years, 7,300 patients had biochemical pregnancy, 6,320 patients had clinical pregnancy, and 5,324 patients achieved a successful live birth. Table 2 presents the relationships between endometrial thickness, AMH, AFC, FSH, and the biochemical pregnancy rate, clinical pregnancy rate, and live birth rate. We observed that an increase in endometrial thickness within the 7–14 mm range, as well as AMH and AFC levels within the relatively normal range, was significantly associated with improved outcomes. For instance, in Model 2, for every 1-mm increment in endometrial thickness, the biochemical pregnancy rate increased by 1.5% [HR 95% CI: 1.015 (1.002, 1.029)], the clinical pregnancy rate increased by 0.8% [HR 95% CI: 1.008 (1.001, 1.015)], and the live birth rate increased by 0.7% [HR 95% CI: 1.007 (1.001, 1.013)]. Moreover, compared to the abnormal AMH group, the normal AMH group exhibited greater increases in these outcomes, with corresponding increments of 5.5% [HR 95% CI: 1.055 (1.002, 1.111)], 7.9% [HR 95% CI: 1.079 (1.020, 1.140)], and 1% [HR: 1.010 (1.002, 1.018)] (Table 2). Conversely, basal FSH did not exhibit a significant association with pregnancy outcomes.

**TABLE 2:** HRs (95% CIs) for the independent associations of endometrial thickness and of AMH, AFC, and FSH levels with pregnancy outcomes (N = 12,071).

Variables	HRs (95% CIs)	​
	Model 1^a^	Model 2^b^
Biochemical pregnancy (n = 7,300)		
Endometrial thickness	1.030 (1.018, 1.042)	1.015 (1.002, 1.029)
AMH		
Abnormal	Ref	Ref
Normal	1.059 (1.006, 1.115)	1.055 (1.002, 1.111)
AFC		
Abnormal	Ref	Ref
Normal	1.005 (1.008, 1.104)	1.069 (1.021, 1.119)
FSH		
Abnormal	Ref	Ref
Normal	1.026 (0.959, 1.096)	1.018 (0.952, 1.090)
Clinical pregnancy (n = 6,320)		
Endometrial thickness	1.018 (1.009, 1.027)	1.008 (1.001, 1.015)
AMH		
Abnormal	Ref	Ref
Normal	1.079 (1.021, 1.140)	1.079 (1.020, 1.140)
AFC		
Abnormal	Ref	Ref
Normal	1.063 (1.012, 1.116)	1.065 (1.014, 1.119)
FSH		
Abnormal	Ref	Ref
Normal	0.994 (0.925, 1.067)	0.993 (0.924, 1.068)
Live births (n = 5,324)		
Endometrial thickness	1.009 (1.002, 1.016)	1.007 (1.001, 1.013)
AMH		
Abnormal Normal	Ref	Ref
	1.013 (1.005, 1.021)	1.010 (1.002, 1.018)
AFC		
Abnormal	Ref	Ref
Normal	1.020 (1.005, 1.036)	1.033 (1.019, 1.048)
FSH		
Abnormal	Ref	Ref
Normal	0.973 (0.900, 1.051)	0.956 (0.883, 1.034)

Abbreviations: CI, confidence interval; AMH, Anti-mullerian hormone; AFC, antral follicle count; FSH, follicle stimulating hormone; Ref, Reference. endometrial thickness indicates the effect of each unit increase on results.

^a^ Model 1 was adjusted for female age and female ethnicity.

^b^ Model 2 added to Model 1 ovarian stimulation protocol, total gonadotropin dose, estradiol level and endometrial pattern on hCG, day, body mass index, infertility duration, prolactin level, number of high-quality cleavage-stage embryos transferred, number of high-quality blastocyst-stage embryos transferred, male sperm concentration, smoking status, blood glucose, triglyceride, total cholesterol, and low-density lipoprotein cholesterol (LDL-C) levels. The proportional hazards assumption of the model was tested using the Schoenfeld residual method, with all covariates satisfying *P* > 0.05, thus meeting the prerequisites for model application.

### 3.3 Effect modification by AMH, AFC, FSH

**FIGURE 3:**
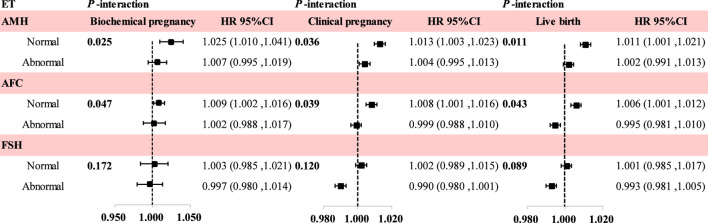
Effect modification by AMH, AFC, and basal FSH on the association between endometrial thickness and pregnancy outcomes. We calculated the risk HR and their corresponding 95% CI associated with each one-unit increase in endometrial thickness across varying levels of AMH, AFC and basal FSH. Stratified analysis was performed using the Cox proportional hazards regression model, based on different levels of AMH, AFC, and basal FSH. The interaction was tested by comparing the log-likelihood values of models with and without interaction terms between AMH, AFC, basal FSH and endometrial thickness. Models were adjusted for female age, female ethnicity, ovarian stimulation protocol, total gonadotropin dose, estradiol level and endometrial pattern on hCG day, body mass index, infertility duration, prolactin level, number of high-quality cleavage-stage embryos transferred, number of high-quality blastocyst-stage embryos transferred, male sperm concentration, smoking status, blood glucose, triglyceride, total cholesterol, and low-density lipoprotein cholesterol (LDL-C) levels.

We investigated the modifying effects of AMH, AFC, FSH, and endometrial thickness on biochemical pregnancy, clinical pregnancy, and live birth (Figure 3). For patients with biochemical pregnancy, we found significant interactions between endometrial thickness and AMH (*P*-interaction = 0.025) and between endometrial thickness and AFC (*P*-interaction = 0.047). Compared with patients having relatively abnormal hormone levels, the association between endometrial thickness and biochemical pregnancy was notably stronger in patients with relatively normal hormone levels. Specifically, in patients within the AMH abnormal response group, the HR (95% CI) for each 1-mm increase in endometrial thickness was 1.007 (0.995, 1.019), while in patients with normal AMH levels, it was 1.025 (1.010, 1.041). In addition, we also detected significant interactions among AMH, AFC, and endometrial thickness regarding clinical pregnancy and live birth (*P*-interaction < 0.05).

### 3.4 Sensitivity analyses

After excluding 280 patients with an infertility duration exceeding 10 years, all the analysis results indicated no substantial alterations (Supplementary Table S1). When we employed multiple imputation to handle the missing data of AMH, AFC, FSH, and endometrial thickness, the results remained essentially unchanged (Supplementary Table S2). Furthermore, adjusting the predefined thresholds did not substantially alter the associations between endometrial thickness and pregnancy outcomes, confirming the robustness of these thresholds (Supplementary Figure S1). Age-stratified analyses revealed that, across both the <35 years (Supplementary Figure S2) and ≥35 years (Supplementary Figure S3) subgroups, the direction of the association between endometrial thickness and pregnancy outcomes was largely consistent across different ovarian response groups, with no significant pattern alterations observed.

## 4 Discussion

In this large-scale retrospective cohort study involving patients undergoing fresh embryo transfer, we observed that among those with an endometrial thickness of 7–14 mm on the day of hCG administration, each 1-mm increase in endometrial thickness was modestly associated with improved pregnancy outcomes, including biochemical pregnancy, clinical pregnancy, and live birth. Further stratified analyses demonstrated that this association was more pronounced in patients with relatively normal levels of AMH and AFC, indicating that ovarian reserve status may modulate the relationship between endometrial thickness and pregnancy outcomes. (*p*-interaction <0.05).

### 4.1 Comparison with prior evidence and possible explanations

Currently, the impact of endometrial thickness on assisted reproductive outcomes remains a subject of extensive debate, with no consensus on a universal threshold for predicting pregnancy outcomes (Schoyer et al., 2025). A multicenter study across 25 reproductive centers in 3 countries reported significantly lower live birth rate (LBR) when endometrial thickness was <7 mm, although the extent of the decline and the specific thickness threshold varied among centers and cycle types (Genovese et al., 2025). Another retrospective cohort study from the Canadian Assisted Reproductive Technology Registry, including 53,377 fresh embryo transfer cycles, observed that LBR increased with endometrial thickness until plateauing at 7–10 mm (Mahutte et al., 2022). Gingold et al., analyzing data from 182,784 patients in the Society for Assisted Reproductive Technology (SART) national registry, found that an increase in endometrial thickness from 5 mm to 8 mm was strongly associated with a significant rise in LBR, a beneficial trend that continued up to 12 mm (Gingold et al., 2025). Xu et al. conducted a retrospective curve-fitting and threshold-effect analysis of 40,000 cycles from 5 reproductive centers in China, which revealed that miscarriage rates increased significantly when endometrial thickness reached ≥15 mm (Xu et al., 2021). The results of the present study further indicate that within the 7–14 mm range, biochemical pregnancy, clinical pregnancy, and live birth rates all showed an upward trend with increasing endometrial thickness.

AMH is a crucial biomarker of ovarian reserve in women of reproductive age (Cedars, 2022). Although AMH has been demonstrated to predict the risk of poor ovarian response or ovarian hyperstimulation syndrome, its utility in predicting ongoing pregnancy remains limited (Moolhuijsen and Visser, 2020). To date, there is no unified view on the interaction between endometrial thickness and AMH. One study provided evidence that the presence of AMH and its receptors in the endometrium suggests that AMH can act on the endometrium through autocrine, paracrine, and endocrine mechanisms (Dewailly et al., 2014). The concentration of AMH in cultured human endometrial stromal cells significantly reduces cell viability and promotes cell apoptosis (Wang et al., 2009). Mohamed et al.'s research data indicated that extremely high AMH levels (>7 ng/mL) do not exert a negative impact on endometrial thickness (Mohamed et al., 2022). Our study adds to this context by finding that in patients with AMH ≥2.59 ng/mL, endometrial thickness showed a significant positive association with IVF pregnancy outcomes. After controlling for multiple complex variables, such as embryo factors, age, and BMI, in patients with relatively normal AMH levels, for each 1-mm increase in endometrial thickness, the corresponding biochemical pregnancy rate, clinical pregnancy rate, and live birth rate all increased significantly (*p*-interaction <0.001).

AFC is a reliable predictor of ovarian response and is strongly correlated with the severity of ovarian hyperstimulation syndrome (OHSS). However, it cannot independently and effectively predict IVF outcomes (Melo et al., 2009; Sun et al., 2020). Nahum et al. reported that patients with AFC >6 had a significantly higher clinical pregnancy rate compared to those with AFC <6 (51% vs. 19%) (Nahum et al., 2001). The 2023 Delphi expert consensus proposed that the minimum AFC associated with the risk of over-response is 18 (Feferkorn et al., 2023). Another study indicated that AFC alone cannot predict the pregnancy outcomes of IVF patients under 40 years of age (Sahmay et al., 2012). Broer’s research suggested that both AFC and AMH have limited predictive value for non-pregnancy (Broer et al., 2009). In our study, we not only evaluated the predictive efficiency of AFC but also identified an interaction between AFC and endometrial thickness (*p*-interaction <0.001). When the AFC of the included patients was within the relatively normal range, with an increase in endometrial thickness, the biochemical pregnancy rate increased by 0.7% [HR 95% CI: 1.007 (1.001, 1.017)], the clinical pregnancy rate increased by 0.8% [HR 95% CI: 1.008 (1.001, 1.019)], and the live birth rate increased by 0.6% [HR 95% CI: 1.003 (1.001, 1.015)].

Early meta-analyses identified FSH as a key predictor of IVF outcomes (van Loendersloot et al., 2010). A serum basal FSH concentration exceeding 10–12 mIU/mL is conventionally considered indicative of diminished ovarian reserve (DOR) (Harris et al., 2023; Toner et al., 1991). In contrast, Barad et al. suggested that DOR may still be likely even when basal FSH falls within the normal range, provided it exceeds the age-specific 95% confidence interval upper limit (Barad et al., 2007). Creus et al. further emphasized that basal FSH is age-dependent and should be interpreted in conjunction with patient age to predict IVF success (Creus et al., 2000). In our study, however, abnormal basal FSH levels showed no significant association with live birth rate compared with normal levels [HR 0.96, 95% CI 0.88–1.03].

Our findings demonstrate that when AMH ≥2.59 ng/mL or AFC ≥11, each 1-mm increase in endometrial thickness is associated with a modest improvement in pregnancy outcomes. Given the strong positive correlation between AMH and AFC (Hochberg et al., 2025; Anderson et al., 2015), it is suggested that these two ovarian markers may exert comparable regulatory effects on endometrial thickness. However, the underlying mechanisms governing their interaction remain elusive. Current literature presents conflicting evidence regarding the relationship between endometrial thickness and ovarian reserve parameters. Sacha et al. observed an inverse correlation between follicular fluid AMH concentrations and endometrial thickness (Sacha et al., 2020), while Gaba et al. reported that in clomiphene citrate (CC) cycles, elevated serum AMH levels in patients with polycystic ovary syndrome (PCOS) were associated with reduced endometrial thickness (Gaba et al., 2019). Conversely, a study of 1,926 gonadotropin-stimulated cycles identified a weak positive correlation between serum AMH levels and endometrial thickness on the day of hCG trigger (Vagios et al., 2023). Wang et al. further demonstrated that elderly women with low AMH and AFC levels exhibited smaller dominant follicle diameters during ovulation, higher resistance index (RI) in the subendometrial region, and lower vascularization index (VI), flow index (FI), and vascularization flow index (VFI) compared to younger counterparts (Wang et al., 2020). These observations suggest that diminished AMH and AFC may impair dominant follicle development, leading to reduced estrogen production and compromised endometrial perfusion, thereby hindering endometrial growth (Lledo et al., 2025). Additionally, Abbassi et al. proposed that AMH might modulate endometrial receptivity via regulation of the endometrial microbiome: patients with low serum AMH levels showed higher microbial growth rates in endometrial cultures, whereas increasing AMH levels correlated with decreased detection of pathogenic flora and improved clinical pregnancy rates (Ersahin et al., 2022). Another prospective cohort study noted that in patients with thin endometrium, higher AMH and AFC levels were associated with reduced need for repeated platelet-rich plasma (PRP) treatments (Abbassi et al., 2024), indirectly supporting potential crosstalk between endometrial characteristics and ovarian reserve markers.

### 4.2 Strengths and limitations

The strengths of this study lie in its systematic analysis of the integrated effect of endometrial thickness and ovarian reserve markers on pregnancy outcomes. This was further enhanced by stratified analyses to examine their interaction effects. This approach contributes to a more nuanced and individualized clinical assessment prior to embryo transfer. The reliability of these association analyses is supported by a sample size of over 12,000 transfer cycles.

However, this study has several limitations. First, although endometrial thickness between 7 and 14 mm showed a positive correlation with pregnancy outcomes, the effect size was small, and its clinical significance may be limited. Furthermore, while we observed an interaction between endometrial thickness and AMH/AFC, the underlying mechanism remains unclear. Our conclusions are based on this study’s data and prior literature; further basic research is needed for validation. Finally, the manual measurement of endometrial thickness and AFC is subject to inter-observer variability. As a retrospective study, it is challenging to completely eliminate such measurement bias. In recent years, automated measurement methods have been proposed to improve objectivity. For example, Liu et al. developed a deep learning-based semantic segmentation method to automatically measure endometrial thickness from transvaginal ultrasound images, which could help reduce human-related measurement errors (Liu et al., 2022). Wang et al. developed an automated method that segments the endometrium and measures its thickness from 3D transvaginal ultrasound (TVUS) images, allowing for automated and precise thickness assessment (Wang et al., 2022). Another study reported that automated follicular diameter measurement using specific 3D software is more accurate than conventional two-dimensional ultrasound for determining AFC (Vanden et al., 2021; Rodrigues et al., 2022). In future research, we could adopt such automated techniques to reduce confounding bias due to measurement variability.

## 5 Conclusion

In summary, among patients undergoing fresh embryo transfer with an endometrial thickness ranging from 7 to 14 mm, each 1-mm increase in endometrial thickness was associated with a modest improvement in biochemical pregnancy rate, clinical pregnancy rate, and live birth rate. This association was more pronounced in patients with AMH levels AMH ≥2.59 ng/mL or AFC ≥11.

## Data Availability

The raw data supporting the conclusions of this article will be made available by the authors, without undue reservation.
