# Supplementary material for: The impact of the interaction between ovarian markers and the endometrium on the outcomes of assisted reproduction
Source: Front Cell Dev Biol. 2026 May 1;14:1725501. doi: 10.3389/fcell.2026.1725501 (PMC13177303; doi:10.3389/fcell.2026.1725501)
Supplement: Supplementary file 1 [file DataSheet1.docx]

**The Impact of the Interaction between Ovarian Markers and the Endometrium on the Outcomes of Assisted Reproduction**

Table S1. HRs (95% CIs) for the independent associations of endometrial thickness and of AMH, AFC, and FSH levels with pregnancy outcomes (excluding 280 patients with infertility duration over 10 years, n = 11,791).

| Variables | HRs (95 % CIs) |  |
| --- | --- | --- |
|  | Model 1^a^ | Model 2^a^ |
| Biochemical pregnancy (n = 7,126) | |  |
| Endometrial thickness | 1.038  (1.026, 1.050) | 1.017  (1.003, 1.031) |
| AMH |  |  |
| Abnormal | Ref | Ref |
| Normal | 1.092  (1.042, 1.133) | 1.061  (1.011, 1.112) |
| AFC |  |  |
| Abnormal | Ref | Ref |
| Normal | 1.123  (1.071, 1.176) | 1.051  (1.021, 1.082) |
| FSH |  |  |
| Abnormal | Ref | Ref |
| Normal | 1.172  (0.961, 1.384) | 1.035  (0.965, 1.106) |
| Clinical pregnancy (n = 6,167) | |  |
| Endometrial thickness | 1.041  (1.029, 1.054) | 1.016  (1.010, 1.023) |
| AMH |  |  |
| Abnormal | Ref | Ref |
| Normal | 1.081  (1.046, 1.122) | 1.087  (1.034, 1.141) |
| AFC |  |  |
| Abnormal | Ref | Ref |
| Normal | 1.189  (1.112, 1.267) | 1.043  (1.016, 1.071) |
| FSH |  |  |
| Abnormal | Ref | Ref |
| Normal | 1.198  (0.972, 1.425) | 1.016  (0.996, 1.026) |
| Live births (n = 5,190) |  |  |
| Endometrial thickness | 1.025  (1.017, 1.033) | 1.005  (1.002, 1.008) |
| AMH |  |  |
| Abnormal  Normal | Ref | Ref |
|  | 1.037  (1.019, 1.055) | 1.007  (1.003, 1.012) |
| AFC |  |  |
| Abnormal | Ref | Ref |
| Normal | 1.118  (1.051, 1.187) | 1.031  (1.012, 1.051) |
| FSH |  |  |
| Abnormal | Ref | Ref |
| Normal | 1.008  (0.992, 1.025) | 0.966  (0.891, 1.042) |

Abbreviations: CI, confidence interval; AMH, Anti-mullerian hormone; AFC, Antral Follicle Count; FSH, Follicle Stimulating Hormone; Ref, Reference. Endometrial thickness indicates the effect of each unit increase on results. ^a^ Model 1 was adjusted for female age and female ethnicity; Model 2 added to Model 1 ovarian stimulation protocol, total gonadotropin (Gn) dosage, estradiol (E_2_) level and endometrial pattern on the day of hCG administration, body mass index (BMI), duration of infertility, prolactin level, number of high-quality cleavage-stage embryos transferred, number of high-quality blastocyst-stage embryos transferred, male sperm concentration, smoking status, blood glucose level, triglyceride level, total cholesterol level, and low-density lipoprotein cholesterol (LDL-C) level. The proportional hazards assumption of the model was tested using the Schoenfeld residual method, with all covariates satisfying *P* > 0.05, thus meeting the prerequisites for model application.

Table S2. HRs (95% CIs) for the independent associations of endometrial thickness and of AMH, AFC, and FSH levels with pregnancy outcomes (included 34 patients with missing endometrial thickness, AMH, AFC, and FSH, n = 12,717).

| Variables | HRs (95 % CIs) |  |
| --- | --- | --- |
|  | Model 1^a^ | Model 2^a^ |
| Biochemical pregnancy (n = 7,715) | |  |
| Endometrial thickness | 1.040  (1.026, 1.055) | 1.019  (1.003, 1.036) |
| AMH |  |  |
| Abnormal | Ref | Ref |
| Normal | 1.094  (1.042, 1.136) | 1.067  (1.017, 1.115) |
| AFC |  |  |
| Abnormal | Ref | Ref |
| Normal | 1.125  (1.070, 1.178) | 1.053  (1.021, 1.085) |
| FSH |  |  |
| Abnormal | Ref | Ref |
| Normal | 1.173  (0.960, 1.386) | 1.038  (0.965, 1.109) |
| Clinical pregnancy (n = 6,672) | |  |
| Endometrial thickness | 1.043  (1.027, 1.058) | 1.019  (1.011, 1.037) |
| AMH |  |  |
| Abnormal | Ref | Ref |
| Normal | 1.083  (1.047, 1.124) | 1.088  (1.034, 1.143) |
| AFC |  |  |
| Abnormal | Ref | Ref |
| Normal | 1.192  (1.113, 1.269) | 1.045  (1.015, 1.074) |
| FSH |  |  |
| Abnormal | Ref | Ref |
| Normal | 1.196  (0.970, 1.425) | 1.018  (0.997, 1.039) |
| Live births (n = 5,619) |  |  |
| Endometrial thickness | 1.027  (1.020, 1.036) | 1.008  (1.001, 1.015) |
| AMH |  |  |
| Abnormal  Normal | Ref | Ref |
|  | 1.038  (1.021, 1.056) | 1.010  (1.002, 1.018) |
| AFC |  |  |
| Abnormal | Ref | Ref |
| Normal | 1.123  (1.052, 1.189) | 1.032  (1.015, 1.052) |
| FSH |  |  |
| Abnormal | Ref | Ref |
| Normal | 1.012  (0.992, 1.033) | 0.962  (0.890, 1.035) |

Abbreviations: CI, confidence interval; AMH, Anti-mullerian hormone; AFC, Antral Follicle Count; FSH, Follicle Stimulating Hormone; Ref, Reference. Endometrial thickness indicates the effect of each unit increase on results. ^a^ Model 1 was adjusted for female age and female ethnicity; Model 2 added to Model 1 ovarian stimulation protocol, total gonadotropin (Gn) dosage, estradiol (E_2_) level and endometrial pattern on the day of hCG administration, body mass index (BMI), duration of infertility, prolactin level, number of high-quality cleavage-stage embryos transferred, number of high-quality blastocyst-stage embryos transferred, male sperm concentration, smoking status, blood glucose level, triglyceride level, total cholesterol level, and low-density lipoprotein cholesterol (LDL-C) level. The proportional hazards assumption of the model was tested using the Schoenfeld residual method, with all covariates satisfying *P* > 0.05, thus meeting the prerequisites for model application.

Figure S1. Sensitivity Analysis with Adjusted Thresholds: Effect Modification by AMH, AFC, and basal FSH on the Endometrial Thickness-Outcome Association.

To test the robustness of the primary thresholds, a sensitivity analysis was performed by lowering the cut-offs for AMH and AFC by 10% (to 2.33 ng/mL and 10 follicles, respectively) and raising the cut-off for basal FSH by 10% (to 9.58 mIU/mL). Hazard ratios (HRs) and 95% confidence intervals (CIs) per 1‑mm increase in endometrial thickness were calculated using Cox proportional hazards models, stratified by the adjusted hormone/follicle levels. The interaction was assessed by comparing models with and without the relevant interaction terms. All models were adjusted for female age, female ethnicity, ovarian stimulation protocol, total gonadotropin dose, estradiol level and endometrial pattern on hCG day, body mass index, infertility duration, prolactin level, number of high-quality cleavage-stage embryos transferred, number of high-quality blastocyst-stage embryos transferred, male sperm concentration, smoking status, blood glucose, triglyceride, total cholesterol, and low-density lipoprotein cholesterol (LDL-C) levels.

Figure S2. Sensitivity Analysis: Effect Modification by AMH, AFC, and basal FSH on the Endometrial Thickness-Outcome Association in Patients Aged < 35 Years (n = 9,891).

Hazard ratios (HRs) and 95% confidence intervals (CIs) for biochemical pregnancy, clinical pregnancy, and live birth per 1‑mm increase in endometrial thickness were estimated using Cox proportional hazards models, stratified by ovarian reserve marker levels. The interaction between endometrial thickness and each marker was assessed by comparing models with and without the corresponding interaction term. All models were adjusted for female age, female ethnicity, ovarian stimulation protocol, total gonadotropin dose, estradiol level and endometrial pattern on hCG day, body mass index, infertility duration, prolactin level, number of high-quality cleavage-stage embryos transferred, number of high-quality blastocyst-stage embryos transferred, male sperm concentration, smoking status, blood glucose, triglyceride, total cholesterol, and low-density lipoprotein cholesterol (LDL-C) levels.

Figure S3. Sensitivity Analysis: Effect Modification by AMH, AFC, and basal FSH on the Endometrial Thickness-Outcome Association in Patients Aged ≥35 Years (n=2180).

Hazard ratios (HRs) and 95% confidence intervals (CIs) for biochemical pregnancy, clinical pregnancy, and live birth per 1‑mm increase in endometrial thickness were estimated using Cox proportional hazards models, stratified by ovarian reserve marker levels. The interaction between endometrial thickness and each marker was assessed by comparing models with and without the corresponding interaction term. All models were adjusted for female age, female ethnicity, ovarian stimulation protocol, total gonadotropin dose, estradiol level and endometrial pattern on hCG day, body mass index, infertility duration, prolactin level, number of high-quality cleavage-stage embryos transferred, number of high-quality blastocyst-stage embryos transferred, male sperm concentration, smoking status, blood glucose, triglyceride, total cholesterol, and low-density lipoprotein cholesterol (LDL-C) levels.
